# Mixtures of xenoestrogens disrupt estradiol-induced non-genomic signaling and downstream functions in pituitary cells

**DOI:** 10.1186/1476-069X-12-26

**Published:** 2013-03-26

**Authors:** René Viñas, Cheryl S Watson

**Affiliations:** 1 Department of Biochemistry & Molecular Biology, University of Texas Medical Branch, Galveston, TX, 77555-0645, USA

## Abstract

**Background:**

Our study examines the effects of xenoestrogen mixtures on estradiol-induced non-genomic signaling and associated functional responses. Bisphenol-A, used to manufacture plastic consumer products, and nonylphenol, a surfactant, are estrogenic by a variety of assays, including altering many intracellular signaling pathways; bisphenol-S is now used as a bisphenol-A substitute. All three compounds contaminate the environment globally. We previously showed that bisphenol-S, bisphenol-A, and nonylphenol alone rapidly activated several kinases at very low concentrations in the GH_3_/B_6_/F_10_ rat pituitary cell line.

**Methods:**

For each assay we compared the response of individual xenoestrogens at environmentally relevant concentrations (10^-15^ -10^-7^ M), to their mixture effects on 10^-9^ M estradiol-induced responses. We used a medium-throughput plate immunoassay to quantify phosphorylations of extracellular signal-regulated kinases (ERKs) and c-Jun-N-terminal kinases (JNKs). Cell numbers were assessed by crystal violet assay to compare the proliferative effects. Apoptosis was assessed by measuring caspase 8 and 9 activities via the release of the fluorescent product 7-amino-4-trifluoromethylcoumarin. Prolactin release was measured by radio-immunoassay after a 1 min exposure to all individual and combinations of estrogens.

**Results:**

Individual xenoestrogens elicited phospho-activation of ERK in a non-monotonic dose- (fM-nM) and mostly oscillating time-dependent (2.5-60 min) manner. When multiple xenoestrogens were combined with nM estradiol, the physiologic estrogen’s response was attenuated. Individual bisphenol compounds did not activate JNK, while nonylphenol did; however, the combination of two or three xenoestrogens with estradiol generated an enhanced non-monotonic JNK dose–response. Estradiol and all xenoestrogen compounds induced cell proliferation individually, while the mixtures of these compounds with estradiol suppressed proliferation below that of the vehicle control, suggesting a possible apoptotic response. Extrinsic caspase 8 activity was suppressed by estradiol, elevated by bisphenol S, and unaffected by mixtures. Intrinsic caspase 9 activity was inhibited by estradiol, and by xenoestrogen combinations (at 10^-14^ and 10^-8^ M). Mixtures of xenoestrogens impeded the estradiol-induced release of prolactin.

**Conclusions:**

In mixtures expected to be found in contaminated environments, xenoestrogens can have dramatic disrupting effects on hormonal mechanisms of cell regulation and their downstream functional responses, altering cellular responses to physiologic estrogens.

## Background

Humans and wildlife do not usually experience XEs as single compounds, and in fact, they probably are exposed to dozens of them simultaneously, if low concentration ranges are considered [1,2]. Newer studies demonstrating that many of these compounds are quite active at very low concentrations necessitate examination of this question to determine if these multiple actions pose a greater health hazard. We have chosen several compounds to study relatively simple multiple exposure scenarios: bisphenol A (BPA); its recently introduced alternative, bisphenol S (BPS); and another ubiquitous environmental estrogen, nonylphenol (NP). Our studies are well-based in our knowledge of their performance as single compounds.

Bisphenol A (BPA) is a well-known endocrine disruptor that has been commercially used since 1957 [3] for the production of consumer plastic goods, the inner lining of metal food cans and drums, as well as the coating of thermal receipt paper [4,5]. Humans are typically exposed to BPA by skin contact and consumption of contaminated food and water that have come in contact with packaging containing BPA, particularly during the heating of plastic containers [6]. A survey by the National Health and Nutrition Examination Survey (NHANES) found levels of BPA to range from 0.4 - 149 μg/L (1.8 - 660 nM) in urine samples from 92.6% of U. S. residents ≥ 6 years of age [7].

*In-vivo* studies have linked the presence of BPA to developmental disruptions including uterine morphological alterations [8], disruptions in gonadotropin releasing hormone signaling [9,10], and increases in the incidence of ovarian cyst-adenomas when administered prenatally to female rats [11,12]. Human epidemiological studies have also associated BPA exposure with decreased sperm viability and mobility [13], recurrent miscarriages [14], as well as ovarian dysfunction and obesity [15]. Extensive review of the literature by expert panels of the National Institute of Health [16] and the National Toxicology Program [17] also highlighted concern for altered prostate, neurodevelopmental, and behavioral outcomes.

The FDA in mid-July 2012 banned the use of BPA in the manufacturing of baby bottles and drinking cups [18]. Increased global regulations such as these have in turn resulted in the synthesis of alternative bisphenol analogues as replacements for BPA [19]. One such analogue is 4,4^′^- dihydroxydiphenyl sulphone, also known as BPS. Replacement of the BPA-carbon for the BPS-sulfur atom in the central position allows the structure to have increased stability against high temperatures and increased resistance to sunlight [20], thus resulting in a less leachable compound compared to BPA [21]. However, if XEs are present even in very small amounts and are potent, they can have effects that mimic or alter responses to physiologic estrogens.

BPS was recently detected in a variety of paper products, including 87% of paper currencies sampled from 21 different countries (geometric mean of 0.029 μg/g) [22]. BPS was detected in 97% of urine samples (n = 31) from residents of Albany, NY in appreciable amounts [geometric mean of 0.299 ng/ml; 1.2 nM] [23]. The structural similarity of BPS to physiologic estrogens and to the known endocrine disruptor BPA originally raised questions over its safety and its endocrine-disrupting potential. Several *in vitro* studies testing the effects of BPS via genomic mechanisms have shown BPS to mimic estrogenic responses [20,24-27]; however, such studies were done at high concentrations unlikely to be leached from BPS-containing products [21]. We very recently demonstrated that BPS is potently estrogenic via non-genomic signaling pathways in the GH_3_/B_6_/F_10_ rat pituitary cell line, including at the low-dose ranges likely to be present in food items and human fluids [28]. This study also demonstrated that BPS can strongly interfere with the signaling actions of the endogenous estrogen, estradiol (E_2_), at *picomolar-* and sub-*picomolar* concentrations, predominantly via membrane-bound estrogen receptor-α (mERα), leading to alterations in functional responses – cell proliferation and prolactin (PRL) release. Previous studies from our group also examined the estrogenic actions of BPA and NP via this rapid signaling pathway with similar consequences on functional endpoints [29-32].

The aim of our present study was to determine if mixtures of XE compounds could cause signaling alterations (i.e. antagonistic or additionally agonistic) on E_2_-induced non-genomic signaling correlated to functional cellular endpoints. To recreate likely real-world scenarios for exposure to mixtures, we compared each compound alone to a tertiary mixture (BPS + BPA + E_2_), and a quaternary mixture (BPS + BPA + NP + E_2_) over wide concentration ranges of the XEs. We examined XE mixtures in combination with a physiologic level of E_2_, as that is the way most organisms will experience them. In addition, we evaluated the effects of these mixtures on MAPK-associated downstream functional endpoints: cell proliferation; apoptosis (caspase 8 and 9 activity); and PRL secretion. Our medium-throughput quantitative plate assays make possible within-assay comparisons between responses to different compounds and their mixtures at a wide range of concentrations.

## Methods

### Cells and culture conditions

The GH_3_/B_6_/F_10_ rat prolactinoma cell line was originally selected on the basis of its naturally high expression of mERα [33-35]. Cells were routinely sub-cultured with phenol red-free Dulbecco’s Modification of Eagle’s Medium (DMEM, high glucose; Mediatech, Herdon,VA) containing 12.5% horse serum (Gibco BRL, Grand Island, NY) and defined supplemented calf and fetal serum (Thermo Fisher, Waltham, MA) at 2.5% and 1.5%, respectively. Cells of passages 10–20 were used for these experiments.

### Quantitative ERK and JNK Phosphorylation assays

A fixed cell-based immunoassay was employed to quantify phospho-activation of ERK (pERK) and JNK (pJNK), as previously developed and described in detail [36]. Cells (10^4^/well) were plated in 96-well plates (Corning Incorporated, Corning, NY) and allowed to attach for 24 hrs. The cells were then cultured in DMEM containing 1% charcoal-stripped (4X) serum for 48 hrs to deprive the cells of serum hormones. Medium was then removed and the cells exposed to individual XEs alone or as mixtures with a physiologic level of E_2_ (10^-9^ M; Sigma-Aldrich, St. Louis, Mo) to assess time- (0-60 min) and concentration-dependent (10^-15^-10^-7^ M) changes (at 5 min). Both the short time points and range in concentrations chosen were based upon recently published studies from our group [30,31,37,38]. The short time points ensure that we are indeed observing a rapid non-genomic activation of ERK and JNK without genomic interferences, while the concentrations are reflective of levels found in the environment. Test compounds were dissolved in ethanol then diluted in DMEM containing 1% charcoal-stripped serum. Vehicle control (V) was 0.001% ethanol in DMEM. To stop mER-initiated signaling, cells were fixed with a 2% paraformaldehyde/0.2% picric acid solution (Fisher Scientific, Pittsburgh, PA) and incubated at 4°C for 48 hrs. The cells were then incubated with phosphate–buffered saline (PBS) containing 0.2% fish gelatin and 0.1% Triton X-100 (Sigma-Aldrich) for 1 hr at room temperature (RT), followed by overnight incubation at 4°C with primary antibodies (Abs) against pERK or pJNK (Cell Signaling Technology, Beverly, MA; 1:500 in PBS/0.2% fish gelatin/0.1% TritonX-100). The cells were then washed with PBS (3X) before biotin-conjugated Ab (Vector Labs, Burlingame, CA) was applied and incubated (1 hr) at RT (1:500 in PBS/0.2% fish gelatin). The cells were again washed in PBS (3X) and incubated with Vectastain ABC-AP solution (Vector Labs; 50 μL/well) for 1 hr at RT, followed by alkaline phosphatase substrate (pNpp solution; 50 μL/well). The plates were incubated in the dark for 30 min at 37°C and the signal for the product of *para-*nitrophenol phosphate (pNpp) (Thermo Scientific, Rockford, IL) breakdown to pNp was read at A_405_ in a model 1420 Wallac microplate reader (Perkin Elmer, Boston, MA). The pNp signal was normalized to cell number, determined by the crystal violet (CV) assay at A_590_, as described in [39].

### Effects on cell numbers

We have previously described this method for estimating cell numbers in detail [40]. Briefly, sub-confluent cells were seeded into 96-well plates that had been coated with poly-D-lysine (5000 cells/well) and allowed to attach overnight. Plating medium was then replaced with DMEM containing 1% 4X charcoal-stripped serum for 48 hrs, then treated with media containing increasing concentrations of individual XEs (10^-15^-10^-7^ M) or mixtures of BPS + BPA + 10^-9^ M E_2_ or BPS + BPA + NP + 10^-9^ M E_2_. After 3 days, cells were fixed (2% paraformaldehyde/0.1% glutaraldehyde in PBS; 50 μl/well) and cell numbers were assessed by CV assay to compare the proliferative effects of XE-mixtures at different concentrations.

### Determination of caspase activities

Caspase-8 and -9 activities were assessed as previously described [40]. Sub-confluent GH_3_/B_6_/F_10_ cells were seeded into 96-well plates (5 × 10^3^/well) and allowed to attach overnight. Treatments began the next day; cells were exposed for 8 hrs to 1 nM E_2_, 10^-14^ M and 10^-8^ M individual XEs, and mixtures in DMEM-1% 4X charcoal-stripped serum; treatment medium was suctioned off and the cells lysed with 50 μL lysis buffer (10 mM Hepes; 2 mM EDTA; 0.1% CHAPS; pH 7.4) to which 1 mM DTT (1:2000, freshly prepared, Sigma-Aldrich) had been added. Plates were then stored at −70°C until assay. Staurosporine [500 nM] (Sigma-Aldrich) dissolved in DMSO was used as a positive control for activation of caspase-8 and -9. The released fluorescent product 7-amino-4-trifluoromethylcoumarin (AFC) was read using a Flexstation 3 spectrofluorometer (Molecular Devices, Sunnyvale, CA) at 400 nm excitation, and 505 nm emission wavelengths.

### Prolactin release

These assay conditions were based on our previous studies [32,38]. Cells (0.5–0.7× 10^6^) were plated into poly-D-lysine-coated 6-well plates overnight and hormone-deprived in DMEM-1% 4X charcoal-stripped serum for 48 hrs. Cells were then pre-incubated for 30 min in DMEM/0.1% BSA and exposed for 1 min to different concentrations of individual XEs alone (10^-15^ -10^-7^ M), or as mixtures with 10^-9^ M E_2_, then centrifuged at 4°C, 350 × *g* for 5 min. The supernatant was collected and stored at −20°C until radioimmunoassay (RIA) for PRL. Cells were then fixed with 1 ml of 2% paraformaldehyde/0.1% glutaraldehyde in PBS, and cell numbers determined via the CV assay. PRL RIA concentrations were determined with a Wizard 1470 Gamma Counter (Perkin Elmer) and normalized to CV values.

### Statistical analysis

Statistical analyses were performed using Sigmaplot version 12.3 (Systat Software Inc). One-way analysis of variance (ANOVA) was applied to the dose- and time-dependent studies to assess the statistical significance of mean values produced by varying exposures. A Holm-Sidak comparison against vehicle control or against E_2_ treatment was used to evaluate significance. The overall α level selected for the statistical analysis was 0.05.

## Results

### Temporal changes in phospho-activation of MAPKs by BPS, BPA, and NP, and their combinations, during a 60 min exposure

**Figure 1:**
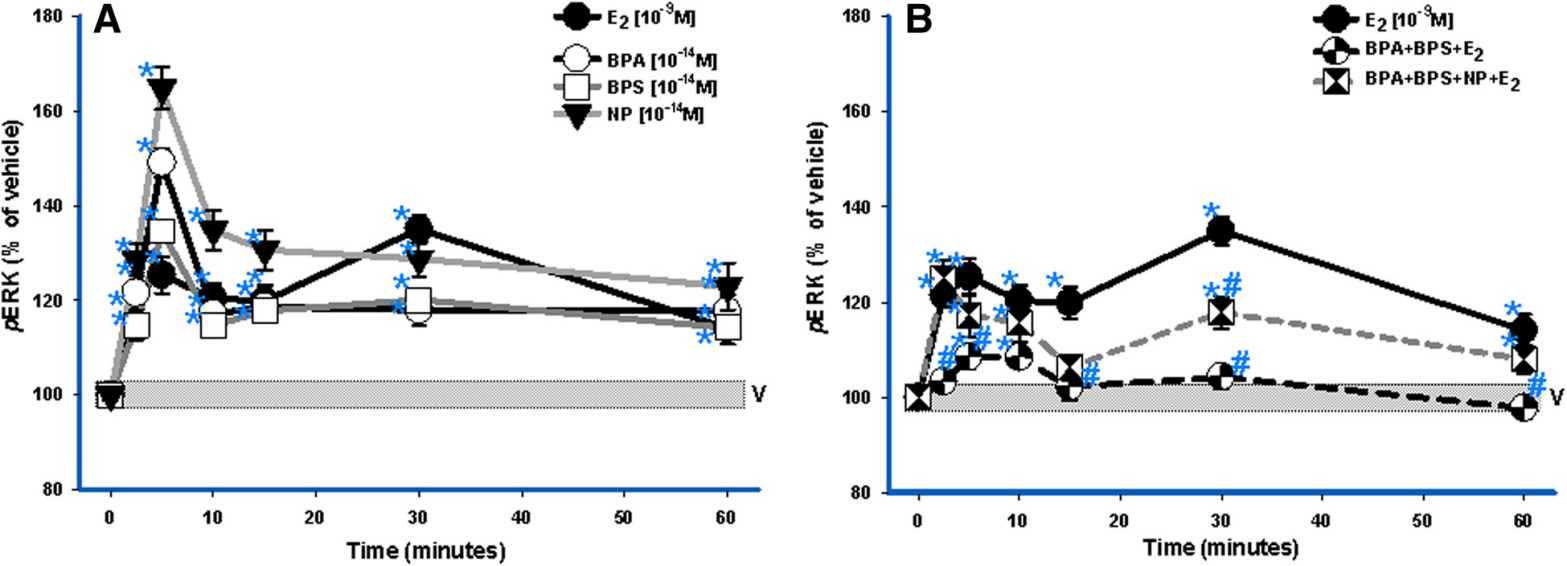
**Timing of ERK activation by E**_**2, **_**XEs, and XE/E**_**2 **_**mixtures.** Rat pituitary cells were exposed to BPS (10^-14^ M), BPA (10^-14^ M), NP (10^-14^ M) and/or E_2_ (10^-9^ M) over a 60- min time course. Responses to individual XEs (**A**) and mixtures (**B**) were measured by plate immunoassay; the pNp signal generated for each well was normalized to cell number (measured by the CV assay). Values are expressed as percentage of vehicle (V)-treated controls. All error bars represent S.E M. The width of the vehicle bar represents a S.E. of ±1.2 (n = 24 over 3 experiments). * = p < 0.05 compared to vehicle (V); **#** = p < 0.05 compared to 10^-9^ M E_2_.

The time dependence of these responses was examined at optimal response concentrations (see Figure 1). E_2_ produced a typical oscillating two-peak ERK response, with the first peak within 5 min, followed by a second peak at 30 min as we have observed previously [31,35-37]. During the same 60 min time frame XEs generated temporal profiles different from E_2_ (Figure 1A). The combination of 10^-14^ M XEs and 10^-9^ M E_2_ (Figure 1B) caused a deviation from the E_2−_induced temporal pattern, as well a decrease of the overall ERK response, as was also seen in the dose-dependent studies (see below). Similar deviations due to other XE combinations with E_2_ have been previously documented [30,31,37,38]. Therefore, even at this very low concentration (10^-14^ M), XEs are capable of disrupting the timing of the response to a physiologic estrogen_._

**Figure 2:**
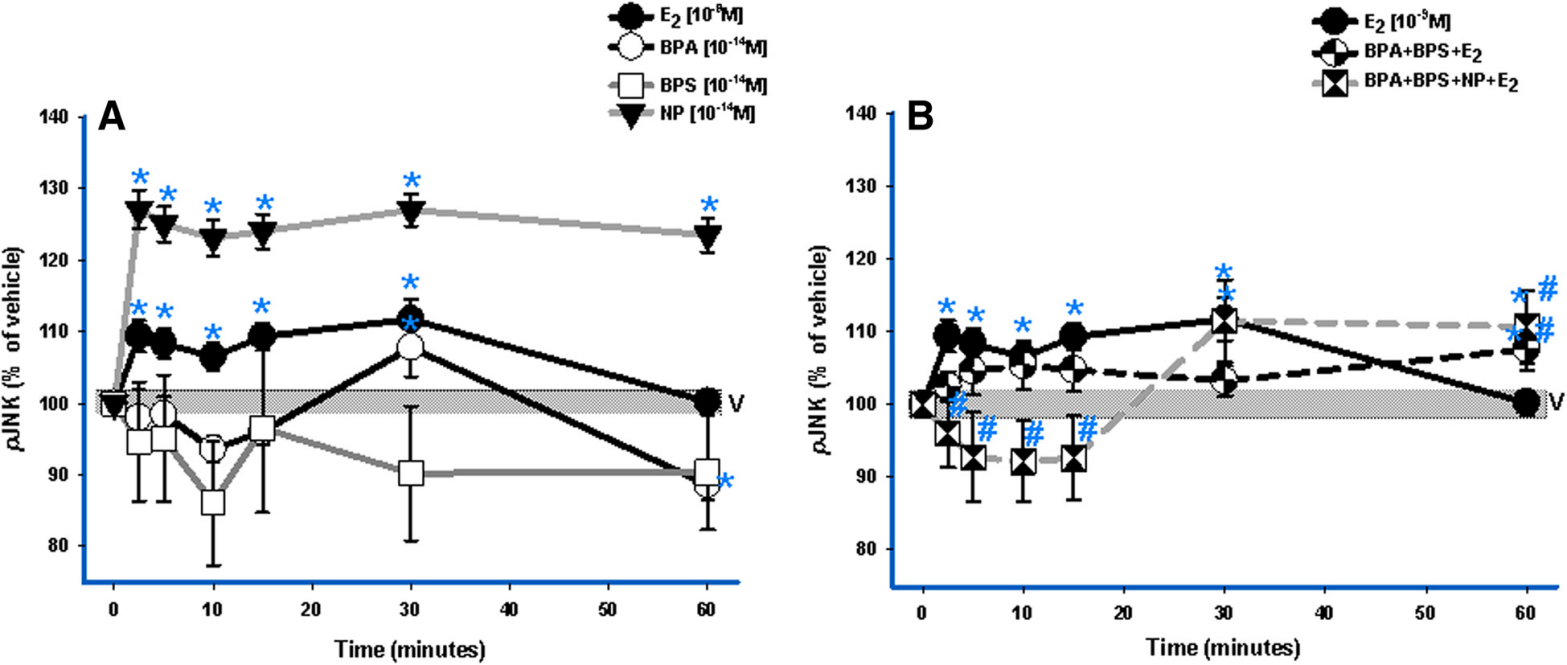
**Timing of JNK activation by E**_**2, **_**XEs, and XE/E**_**2 **_**mixtures.** Rat pituitary cells were exposed to BPS (10^-14^ M), BPA (10^-14^ M), NP (10^-14^ M) and/or E_2_ (10^-9^ M) over a 60- min time course. Responses to individual XEs (**A**) and mixtures (**B**) were measured by plate immunoassay; the pNp signal generated for each well was normalized to cell number (measured by the CV assay). Values are expressed as percentage of vehicle (V)-treated controls. All error bars represent S.E M. The width of the vehicle bar represents a S.E. of ± 1.0 (n = 24 over 3 experiments). * = p < 0.05 compared to vehicle (V); **#** = p < 0.05 compared to 10^-9^ M E_2_.

Neither BPA nor BPS could maintain activation of JNK by themselves during the 60-min time course (Figure 2A); however, their combination with E_2_ (3-compound mixture) did activate JNK with a significant 60-min response, by which time the response to E_2_ had declined to control values (Figure 2B). As a 4-compound mixture, E_2_ plus all XEs inactivated JNK to below vehicle control values early in the time course, but then activated and sustained pJNK after 30 min. Overall, these combinations with XEs markedly attenuated the E_2_-induced JNK response.

### Concentration-dependent changes in phospho-activation of MAPKs by a short exposure to BPS, BPA, and NP, and their combinations

**Figure 3:**
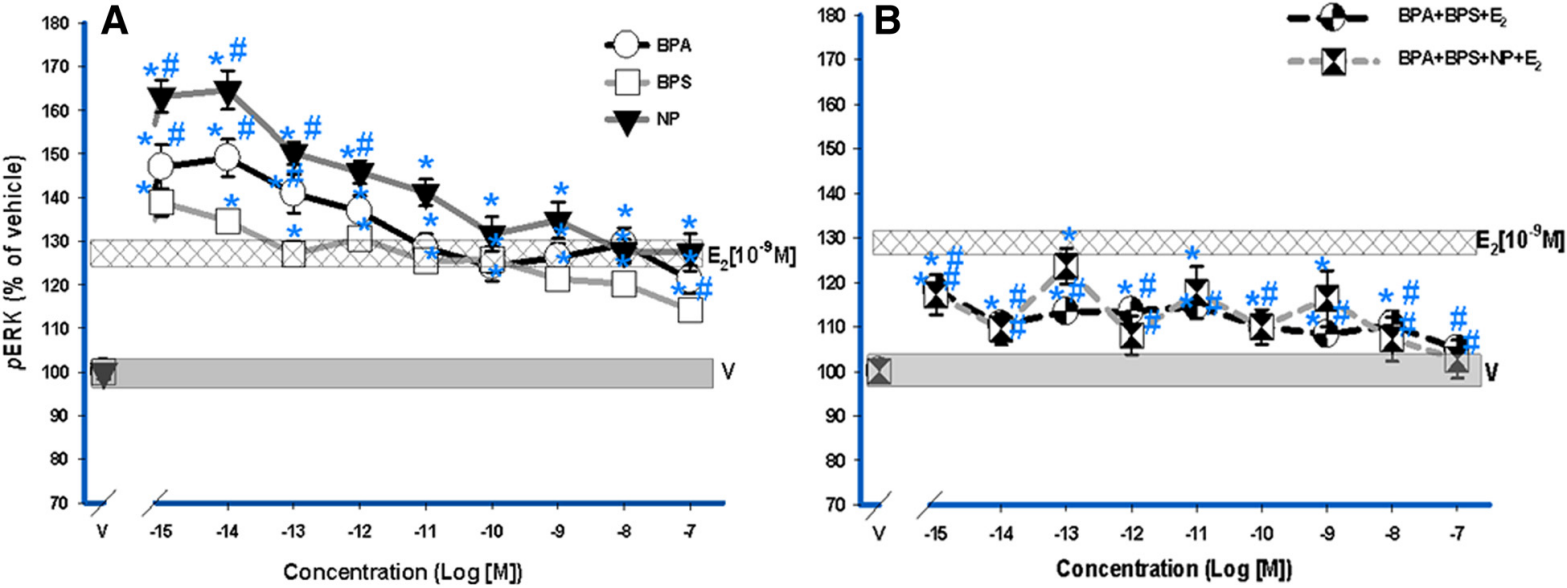
**ERK activation dose-response analysis by E**_**2, **_**XEs, and mixtures.** GH_3_/B_6_/F_10_ rat pituitary cells were exposed to increasing concentrations (10^-15^ M – 10^-7^ M) of BPS, BPA, and NP, compared to a single physiological level of E_2_ (10^-9^ M). E_2_ (10^-9^ M) is at a constant concentration throughout the XE dose-response range. Individual XEs (**A**) and XE mixture responses (**B**) were measured by plate immunoassay at a 5-min exposure time. All error bars represent S.E M. The widths of the vehicle and E_2_ [10^-9^ M] bars represent a S.E. of ± 1.5 and ± 1.2 respectively for both **A** and **B**, (n = 24 over 3 experiments). * = p < 0.05 compared to vehicle (V); **#** = p < 0.05 compared to 10^-9^ M E_2_. The E_2_ (10^-9^ M) response is significantly different compared to the vehicle control.

We have previously determined dose–response profiles for BPS [28], and they are described here for comparison. Short exposures (5 min) to individual XEs (Figure 3A) caused ERK activation in GH_3_/B_6_/F_10_ cells at concentrations similar to those elicited by E_2_[30,31]. The lowest tested XE concentrations evoked a higher pERK response than did 10^-9^ M E_2_. The responses steadily decreased with increasing XE concentrations. Responses to femtomolar concentrations of individual XEs were statistically different (by one-way ANOVA) from those in the nanomolar range and from the zero concentration point, indicating a non-monotonic dose–response [41]. The combination of XEs of increasing concentrations with constant 10^-9^ M E_2_ (Figure 3B) reduced ERK activity below that of either E_2_ or XEs alone, reaching vehicle control levels at the highest concentrations.

**Figure 4:**
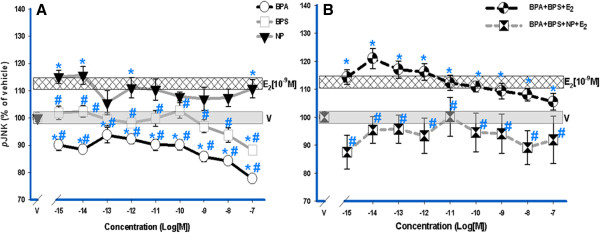
**JNK activation dose-response analysis by E**_**2, **_**XEs, and mixtures.** GH_3_/B_6_/F_10_ rat pituitary cells were exposed to increasing concentrations (10^-15^ M – 10^-7^ M) of BPS, BPA, and NP compared to a single physiological level of E_2_ (10^-9^ M). E_2_ (10^-9^ M) is at a constant concentration throughout the dose-response. Individual XEs (**A**) and XE mixtures (**B**) were measured by plate immunoassay at a 5-min exposure time. All error bars represent S.E M. The widths of the vehicle and E_2_ [10^-9^ M] bars represent a S.E. of ± 1.3 and ± 1.2 respectively, (n = 24 over 3 experiments). * = p < 0.05 compared to vehicle (V); **#** = p < 0.05 compared to 10^-9^ M E_2_. The E_2_ (10^-9^ M) response is significantly different compared to the vehicle control.

Individual bisphenol compounds deactivated pJNK below vehicle levels (Figure 4A), unlike E_2_ and NP that both activated JNK. However, when E_2_ was administered together with both bisphenol compounds (Figure 4B), JNK was strongly activated, featuring a non-monotonic dose–response curve with the lowest concentrations evoking the largest responses; the 4-compound mixture evoked no activation of JNK and was consistently, though not statistically, below the level of the response to vehicle, thus erasing the response to 1 nM E_2_.

### XEs and mixtures affect cell proliferation

**Figure 5:**
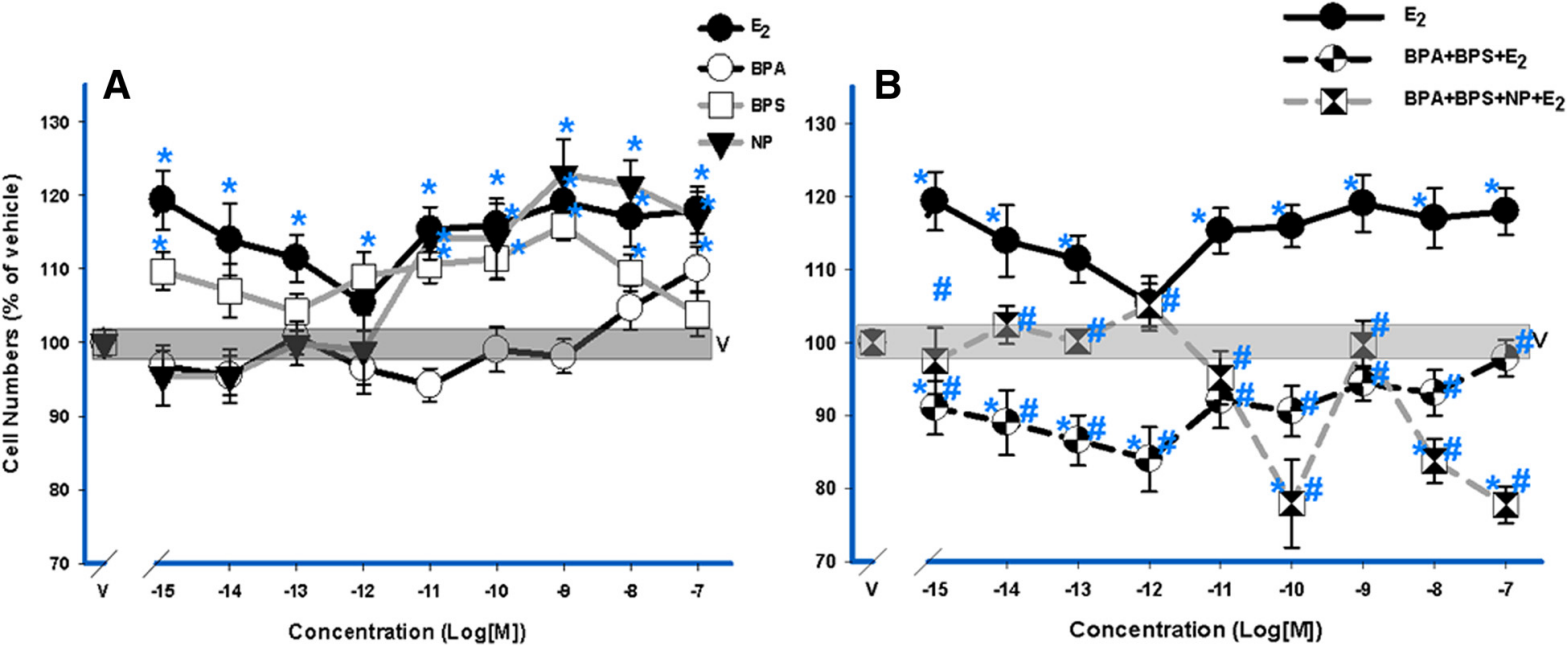
**XEs cause cell proliferation, and XE mixtures disrupt cell proliferation evoked by E**_**2**_**.** Increasing concentrations of XEs (10^-15^ M – 10^-7^ M) compared to increasing concentrations of E_2_ (10^-15^ M-10^-7^ M) alone (**A**) were assessed after a 3-day growth period. Mixtures of E_2_ with XEs were assessed in **B**. Cell number was measured by the CV assay and compared to vehicle (V)-treated cells (n = 24 over 3 experiments). All error bars represent S.E M. The width of the vehicle bar represents a S. E. of ± 1.3. * = p < 0.05 compared to vehicle; in **B**, **#** = p < 0.05 compared to 10^-9^ M E_2_.

After a 3-day exposure, 10^-9^ M E_2_ and BPS had similar effects on cell proliferation [28]. We now looked at the dose responsiveness at this 3-day time point, demonstrating non-monotonic stimulations (Figure 5A), as we observed previously with E_2_ and other XEs [38,40]. NP did not increase cell numbers significantly compared to vehicle until it reached 10^-11^ M, and BPA until it reached 10^-7^ M. Both XE mixtures with E_2_ (Figure 5B) failed to stimulate cell proliferation, but instead suppressed cell numbers far below those seen with vehicle, again showing these compounds’ ability to disrupt a response to a physiologic estrogen.

### Caspases activated and deactivated

**Figure 6:**
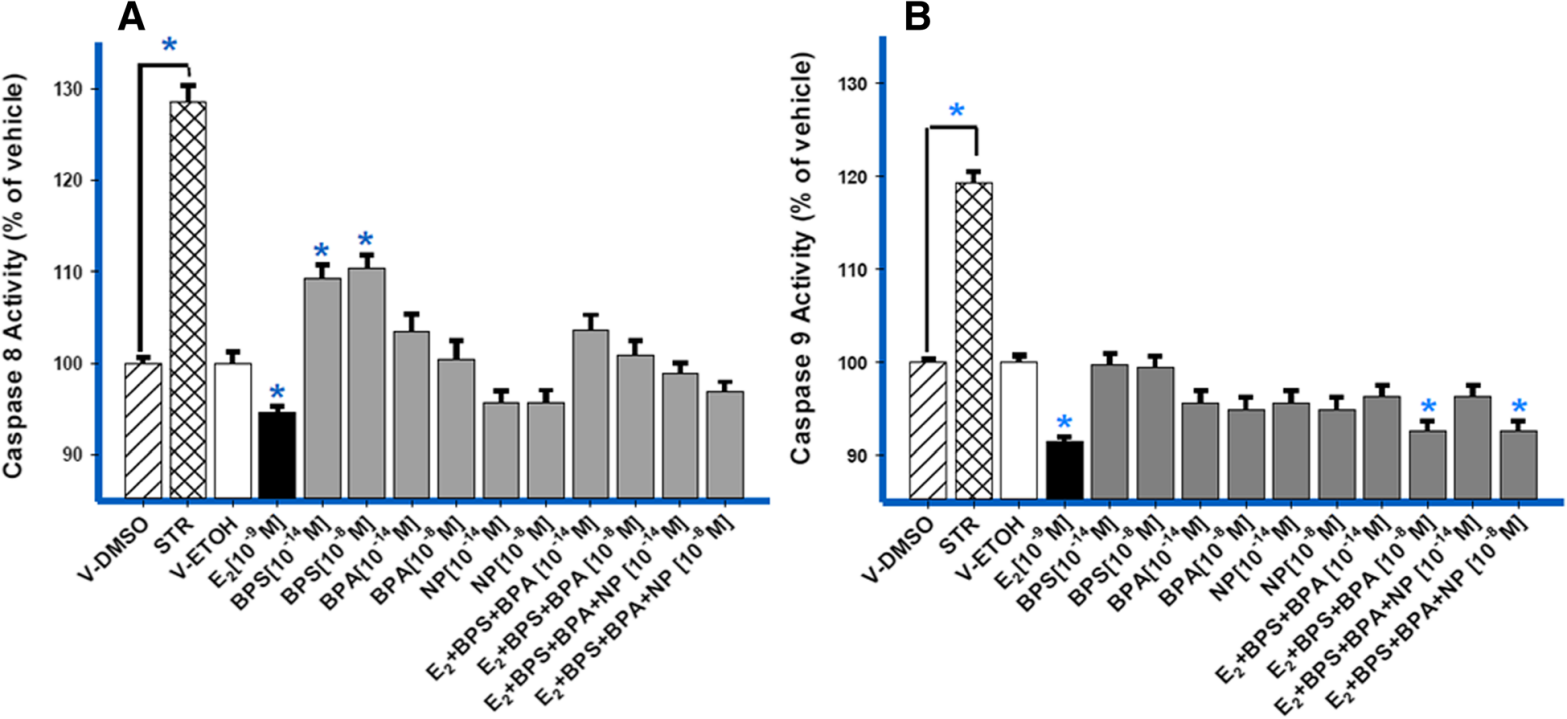
**Activation or deactivation of caspases 8 and 9 by E**_**2, **_**XEs, and mixtures.** Over an 8-hr exposure period we measured caspase 8 (**A**) and 9 (**B**) activity evoked by two different concentrations of BPS, BPA, and NP (10^-14^; 10^-8^ M) separately and together, with each other and with a physiological level of E_2_ (10^-9^ M). E_2_ (10^-9^ M) is at a constant concentration throughout. Caspase activity was measured by the release of a fluorogenic product (AFC) expressed as the percentage of vehicle (V)-treated controls. Staurosporine (STR, 500nM) was used as a positive control for induction of caspase activities compared to its own DMSO V control (n = 24 over 3 experiments). Error bars are means ± S.E. * = p < 0.05 compared to V.

Initiation of apoptosis is one of several factors that can influence cell numbers; we therefore assayed caspase 8 and 9 activities to determine if the extrinsic or intrinsic apoptotic pathways were activated over an 8-hr exposure period, the optimum time that was determined previously [28]. Caspase 8 was significantly activated by BPS at both concentrations used (10^-14^ M and 10^-8^ M), while BPA, NP, and the mixture solutions at their respective concentrations did not result in significant activations (Figure 6A). Activations of caspase 9 were not detected with either individual XEs or mixtures, indicating that the extrinsic pathway (caspase 8) and not the intrinsic pathway (caspase 9) is the primary apoptotic pathway activated. However, both mixture combinations at the highest concentrations (10^-8^ M) resulted in a significant deactivation of caspase 9 activities (Figure 6B). Staurosporine, the positive control for activation, was active on both caspases, as expected. E_2_ by itself suppressed caspase activity below vehicle controls for both apoptotic pathways, as we had seen previously [28,40].

### Mixtures of XEs disrupt E_2_-induced PRL release

**Figure 7:**
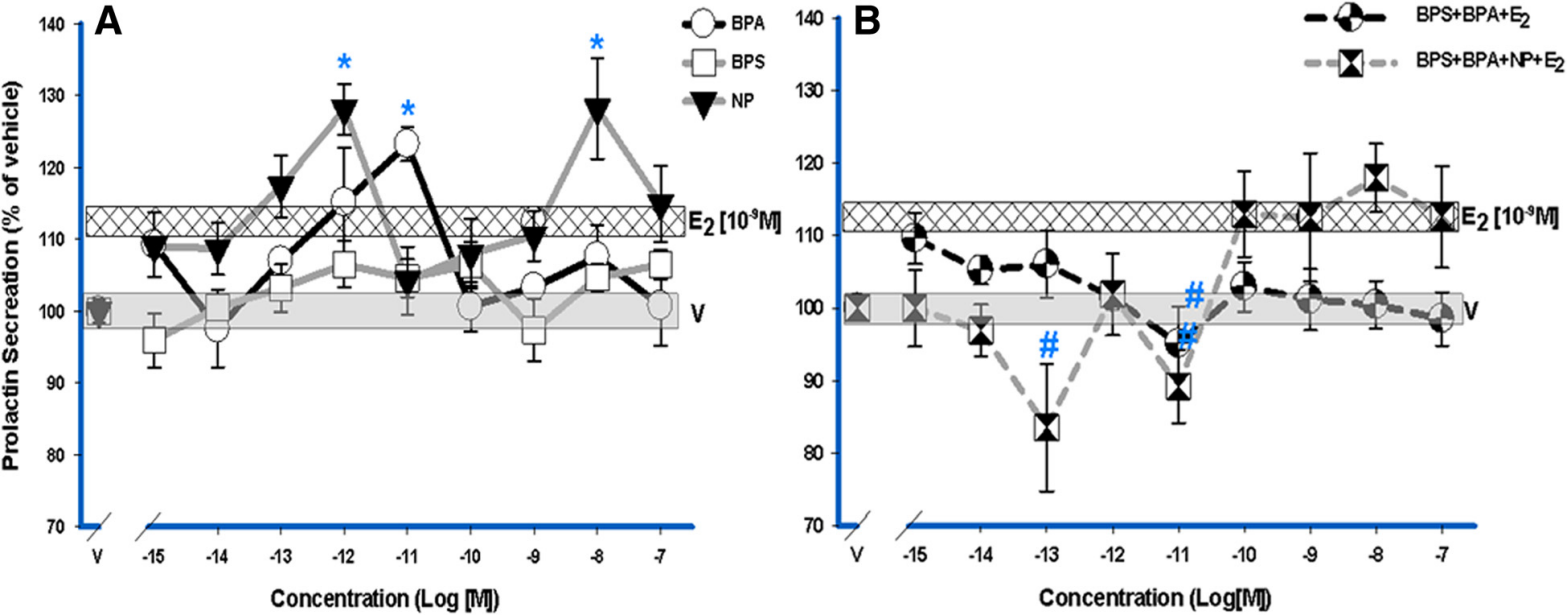
**XEs cause PRL release, and XE mixtures alter these responses.** We measured PRL release into the culture medium by RIA after a 1-min exposure to (**A**) individual XEs (10^-15^ M – 10^-7^ M) and also as (**B**) XE mixtures (10^-15^ M – 10^-7^ M) with a constant physiologic E_2_ concentration (10^-9^ M) throughout the dose-response range of the XEs. The amount of PRL secreted for each well was normalized to the CV value for cell number, and expressed as a percentage of vehicle (V)-treated controls. Error bars are means ± S.E. For positive (E_2_) and negative (V) controls, the width of the bars indicate error ranges (V ±1.5; 10^-9^ M E_2_ ± 1.6). n = 24 over 3 experiments. * = p < 0.05 compared to vehicle; in **B**, **#** = p < 0.05 compared to 10^-9^ M E_2_. The E_2_ (10^-9^ M) response is significantly different compared to the vehicle control.

The rapid non-genomic secretion response for PRL caused by estrogenic exposure in GH_3_/B_6_/F_10_ cells has become a standard tool in our lab for evaluating functional endpoints [30-32,38,42,43]. After a 1-min exposure, BPS could not increase PRL secretion as did E_2_ (Figure 7A [28]). At certain concentrations, BPA and NP were able to significantly increase PRL release, even above that caused by nM E_2_ (Figure 7A); the shape of these dose–response curves are non-monotonic (as confirmed by determining that values at the peaks of activation were statistically different than those at other, usually higher, concentrations). As XE mixtures with 10^-9^ M E_2_, the 3-compound mixture inhibited E_2_-induced PRL release at low concentrations (significantly at 10^-11^ M). The 4-compound mixture caused more extreme inhibitions, even below the vehicle level at the lower concentrations (Figure 7B). Though the 4-compound mixture at 10^-8^ M appears to have resulted in PRL release, the errors in these mixture measurements did not allow this response to be distinguished as statistically different from vehicle, and the mixed signaling patterns caused by the multiple ligands may contribute to this variability.

## Discussion

Our study confirms that the novel BPA substitute, BPS, can initiate rapid non-genomic signaling in pituitary cells at environmentally relevant concentrations (as low as *femto*molar-*pico*molar), as do the more thoroughly tested BPA and NP. In combination these compounds altered endocrine responses differently, and more dramatically. Together, these compounds also interfered with the actions of the physiologic estrogen E_2_ resulting in alterations to functional endpoints. These results not only highlight the need for stricter regulatory requirements for XEs, but also address the need to identify potentially adverse interactions of new chemicals with already existing chemicals in the environment. Such endocrine-disrupting effects should be identified during the initial phases of product development so that hazardous new combination exposures can be prevented [44].

We previously determined that BPS, BPA, and NP had similar high potencies, compared to E_2_, for initiating the phospho-activation of ERK and JNK across a wide range of concentrations and times [28,31,32,37,38,40,45]. Non-monotonic dose–response curves were seen, as low concentrations of individual XEs produced high MAPK activation, decreasing as concentrations increased. The occurrence of non-monotonic responses is the source of much debate. In regards to our system, such occurrences could be due to: 1) negative feedback regulation of MAPKs as concentrations increase, thereby preventing unnecessary pathway activation; 2) receptor down-regulation or desensitization; 3) the presence of multiple receptor subtypes that bind to the same ligand yet initiate interactions with different signaling partners, thereby eliciting different response patterns (stimulatory or inhibitory); and/or 4) the activation of multiple pathways from the same receptor, where signaling can be redundant or divergent [41,46-49]. The more detailed mechanisms responsible for these non-monotonic responses are still largely unexplored at the cellular level.

The phospho-activation of ERK and JNK is often associated with opposing functional endpoints. ERK signaling promotes cell growth and differentiation by activating pro-survival enzymes [50] and inhibiting apoptotic enzymes such as caspases [51-53]. Conversely, JNK signaling is often associated with inflammation or the initiation of cell death, and activating pro-apoptotic proteins (including Bax, caspase-3, Fas, cyclin D1 and under some circumstances, interleukin 1) [54-58]. Our data have shown a correlation between the increase in cell numbers and ERK activation by BPS, as we discussed previously [28]. However, BPA and NP were slightly more efficacious than BPS at activating ERK, yet were unable to increase cell numbers as effectively, suggesting that pro-apoptotic proteins may also be involved in regulating final cell number outcomes. Dramatic decreases in cell numbers, in particular with the 4-compound mixture, could be due to the attenuation of the E_2_-induced ERK activation, as ERK activations are usually linked to cell proliferation responses. Our present data therefore present another example of how the final cell number outcome is dependent on the overall balance between ERK and JNK related activities [59,60].

Caspase activation by XE mixtures was also examined to determine whether activation occurred via an external stimulus (caspase 8) or through an internal stimulus (caspase 9), and to correlate caspase activity to changes in cell numbers. We previously reported that BPS at two concentrations (10^-14^ M and 10^-8^ M) throughout a 4–24 hr time course resulted in activation of caspase 8, with a delayed and probably secondary activation of caspase 9 [28]. However, here we show that BPA, NP, and their mixtures did not activate, and in some cases (as when combined with 10^-9^ M of E_2_), deactivated caspases. Deactivation of caspase activity protects cells from death and contributes to E_2_’s well-known proliferative effect on the GH_3_ cell lines [38,40,61] and other cancer and normal cells. Caspases also contribute to the inhibition of cell proliferation by XE mixtures, but cell numbers are clearly not controlled by caspases alone. The balance of multiple factors – including the actions of several pro-apoptotic or anti-apoptotic enzymes and other proteins – may contribute, along with numerous other proteins that control the cell cycle. However, it is clear that XEs can alter such responses.

A broad range of BPA and NP concentrations induced significant PRL secretion, with some compounds producing non-monotonic dose responses, agreeing with previous studies from our lab [32,38]. However, mixtures of BPS with BPA, and additionally NP, disrupted E_2_-induced PRL release, causing an overall attenuation of secretion compared to individual XE compounds. Such inhibitory actions could be part of a negative feedback mechanism protecting against excess stimulation by multiple estrogens causing unnecessary PRL release. Because PRL regulates over 300 biological functions directly and indirectly [62], alteration of its secretion (either enhancement or inhibition) can cause many different physiologic complications, including such medical problems as metabolic dysfunctions, behavioral disturbances, or reproductive and offspring-rearing failures.

We cannot know for sure if effects in cells and even animal models represent what will happen to humans, because humans usually will not manifest these exposure-based adverse health effects for many years. BPS has only been present in products and the environment for a short time, and therefore, the epidemiology results will not be available for some time to come. In real-world situations, environmental and even occupational exposures are rarely due to a single chemical, but instead involve complex chemical mixtures. Potential health hazards from mixtures are a challenge for regulatory agencies to evaluate, due to the difficulty of testing a vast number of chemical interactions that target various mechanisms and that can be tissue-dependent; these evaluations also require the testing of ever-increasing numbers of chemicals. These quantitative approaches should also contribute to the ability of any regulatory agency to systematically study the interactions of any combinations of compounds including contaminants, drug-drug interactions and drug-pollutant interactions in the drug development process [63].

## Conclusions

Adverse actions from chemicals introduced to the environment should be suspected whenever they can disrupt the actions of a physiologic hormone like E_2_. In addition, compounds acting as estrogens on their own and possibly causing estrogenic responses in an organism at inappropriate life stages have been shown in many studies to be unfavorable. As growing concern over the safety of BPA has led to stricter regulatory actions, we are likely to see other chemical replacements offered. The increased presence of BPS in an environment already contaminated with BPA, NP, and a variety of other prevalent and persistent environmental pollutants now requires increasing scrutiny of their potential hazards as chemical mixtures, and especially those that act via ERs [31,36,64]. Our tissue-relevant responses, such as the ones we have demonstrated with these medium -throughput quantitative assays in pituitary cells, offer efficient test systems that could be used to monitor pollutant mixtures at the cellular level. In addition, altered pituitary responses are very likely to have consequences for all other endocrine tissues. However, it is important to establish effective pre-screening of the endocrine-disruptive potential of any new chemicals whose structures make them candidates for these endocrine-disruptive activities in the future [44].

## Abbreviations

Ab: Antibody; BPA: Bisphenol A; BPS: Bisphenol S; JNK: Jun N terminal kinase; CV: Crystal violet; DMEM: Dulbecco’s Modified Eagle Medium; ERK: Extracellular signal regulated kinase; E2: Estradiol; ER: Estrogen receptor; mERα: Membrane estrogen receptor α; mERβ: Membrane estrogen receptor β; MAPKs: Mitogen activated protein kinases; NP: Nonylphenol; pERK: Phosphorylated ERK; pJNK: Phosphorylated JNK; PRL: Prolactin; XE: Xenoestrogen.

## Competing interests

The authors declare that they have no competing interests.

## Authors’ contributions

RV carried out the experiments on the effects of chemical mixtures on kinase-activation, proliferation, caspase activity, and PRL release studies. All authors participated in the design and analyses of the studies, and wrote, read, and approved the final manuscript.

## References

[B1] Cariot A Dupuis A Albouy-Llaty M Legube B Rabouan S Migeot V Reliable quantification of bisphenol A and its chlorinated derivatives in human breast milk using UPLC-MS/MS method Talanta 2012 100 175 182 23141326 10.1016/j.talanta.2012.08.034

[B2] Woodruff TJ Zota AR Schwartz JM Environmental chemicals in pregnant women in the United States: NHANES 2003–2004 Environ Health Perspect 2011 119 878 885 10.1289/ehp.1002727 21233055 PMC3114826

[B3] Bisphenol A Global Industry Group Bisphenol A: Information Sheet http://bisphenol-a.org/pdf/DiscoveryandUseOctober2002.pdf

[B4] Zalko D Jacques C Duplan H Bruel S Perdu E Viable skin efficiently absorbs and metabolizes bisphenol A Chemosphere 2011 82 424 430 10.1016/j.chemosphere.2010.09.058 21030062

[B5] Willhite CC Ball GL McLellan CJ Derivation of a bisphenol A oral reference dose (RfD) and drinking-water equivalent concentration J Toxicol Environ Health B Crit Rev 2008 11 69 146 10.1080/10937400701724303 18188738

[B6] Kubwabo C Kosarac I Stewart B Gauthier BR Lalonde K Lalonde PJ Migration of bisphenol A from plastic baby bottles, baby bottle liners and reusable polycarbonate drinking bottles Food Addit Contam Part A Chem Anal Control Expo Risk Assess 2009 26 928 937 10.1080/02652030802706725 19680968

[B7] Calafat AM Ye X Wong LY Reidy JA Needham LL Exposure of the U.S. population to bisphenol A and 4-tertiary-octylphenol: 2003–2004 Environ Health Perspect 2008 116 39 44 18197297 10.1289/ehp.10753 PMC2199288

[B8] Berger RG Foster WG De Catanzaro D Bisphenol-A exposure during the period of blastocyst implantation alters uterine morphology and perturbs measures of estrogen and progesterone receptor expression in mice Reprod Toxicol 2010 30 393 400 10.1016/j.reprotox.2010.06.006 20599497

[B9] Fernandez M Bianchi M Lux-Lantos V Libertun C Neonatal exposure to bisphenol A alters reproductive parameters and gonadotropin releasing hormone signaling in female rats Environ Health Perspect 2009 117 757 762 19479018 10.1289/ehp.0800267 PMC2685838

[B10] Abraham IM Han SK Todman MG Korach KS Herbison AE Estrogen receptor beta mediates rapid estrogen actions on gonadotropin-releasing hormone neurons in vivo J Neurosci 2003 23 5771 5777 12843281 10.1523/JNEUROSCI.23-13-05771.2003 PMC6741236

[B11] Fernandez M Bourguignon N Lux-Lantos V Libertun C Neonatal exposure to bisphenol A and reproductive and endocrine alterations resembling the polycystic ovarian syndrome in adult rats Environ Health Perspect 2010 118 1217 1222 10.1289/ehp.0901257 20413367 PMC2944080

[B12] Newbold RR Jefferson WN Padilla-Banks E Prenatal exposure to bisphenol A at environmentally relevant doses adversely affects the murine female reproductive tract later in life Environ Health Perspect 2009 117 879 885 19590677 10.1289/ehp.0800045 PMC2702400

[B13] Li DK Zhou Z Miao M He Y Wang J Ferber J Herrinton LJ Gao E Yuan W Urine bisphenol-A (BPA) level in relation to semen quality Fertil Steril 2011 95 625 630 10.1016/j.fertnstert.2010.09.026 21035116

[B14] Sugiura-Ogasawara M Ozaki Y Sonta S Makino T Suzumori K Exposure to bisphenol A is associated with recurrent miscarriage Hum Reprod 2005 20 2325 2329 10.1093/humrep/deh888 15947000

[B15] Takeuchi T Tsutsumi O Ikezuki Y Takai Y Taketani Y Positive relationship between androgen and the endocrine disruptor, bisphenol A, in normal women and women with ovarian dysfunction Endocr J 2004 51 165 169 10.1507/endocrj.51.165 15118266

[B16] Richter CA Birnbaum LS Farabollini F Newbold RR Rubin BS Talsness CE Vandenbergh JG Walser-Kuntz DR vom Saal FS In vivo effects of bisphenol A in laboratory rodent studies Reprod Toxicol 2007 24 199 224 10.1016/j.reprotox.2007.06.004 17683900 PMC2151845

[B17] Chapin RE Adams J Boekelheide K Gray LE Jr Hayward SW Lees PS McIntyre BS Portier KM Schnorr TM Selevan SG Vandenbergh JG Woskie SR NTP-CERHR expert panel report on the reproductive and developmental toxicity of bisphenol A Birth Defects Res B Dev Reprod Toxicol 2008 83 157 395 10.1002/bdrb.20147 18613034

[B18] Food and Drug Administration: Indirect Food Additives: Polymers. Docket No. FDA-2012-F-0031 http://www.gpo.gov/fdsys/pkg/FR-2012-07-17/pdf/2012-17366.pdf

[B19] Gallar-Ayala H Moyano E Galceran MT Analysis of bisphenols in soft drinks by on-line solid phase extraction fast liquid chromatography-tandem mass spectrometry Anal Chim Acta 2011 683 227 233 10.1016/j.aca.2010.10.034 21167975

[B20] Kuruto-Niwa R Nozawa R Miyakoshi T Shiozawa T Terao Y Estrogenic activity of alkylphenols, bisphenol S, and their chlorinated derivatives using a GFP expression system Environ Toxicol Pharmacol 2005 19 121 130 10.1016/j.etap.2004.05.009 21783468

[B21] Vinas P Campillo N Martinez-Castillo N Hernandez-Cordoba M Comparison of two derivatization-based methods for solid-phase microextraction-gas chromatography–mass spectrometric determination of bisphenol A, bisphenol S and biphenol migrated from food cans Anal Bioanal Chem 2010 397 115 125 10.1007/s00216-010-3464-7 20127078

[B22] Liao C Liu F Kannan K Bisphenol s, a new bisphenol analogue, in paper products and currency bills and its association with bisphenol a residues Environ Sci Technol 2012 46 6515 6522 10.1021/es300876n 22591511

[B23] Liao C Liu F Alomirah H Loi VD Mohd MA Moon HB Nakata H Kannan K Bisphenol s in urine from the United States and seven Asian countries: occurrence and human exposures Environ Sci Technol 2012 46 6860 6866 10.1021/es301334j 22620267

[B24] Grignard E Lapenna S Bremer S Weak estrogenic transcriptional activities of Bisphenol A and Bisphenol S Toxicol In Vitro 2012 26 727 731 10.1016/j.tiv.2012.03.013 22507746

[B25] Hashimoto Y Nakamura M Estrogenic activity of dental materials and bisphenol-A related chemicals *in vitro* Dent Mater J 2000 19 245 262 10.4012/dmj.19.245 11218845

[B26] Hashimoto Y Moriguchi Y Oshima H Kawaguchi M Miyazaki K Nakamura M Measurement of estrogenic activity of chemicals for the development of new dental polymers Toxicol In Vitro 2001 15 421 425 10.1016/S0887-2333(01)00046-7 11566573

[B27] Chen MY Ike M Fujita M Acute toxicity, mutagenicity, and estrogenicity of bisphenol-A and other bisphenols Environ Toxicol 2002 17 80 86 10.1002/tox.10035 11847978

[B28] Vinas R Watson CS Bisphenol s disrupts estradiol-induced nongenomic signaling in a rat pituitary cell line: effects on cell functions Environ Health Perspect 2013 121 3 352 358 10.1289/ehp.1205826 23458715 PMC3621186

[B29] Alyea RA Watson CS Nongenomic mechanisms of physiological estrogen-mediated dopamine efflux BMC Neurosci 2009 10 59 10.1186/1471-2202-10-59 19531209 PMC2708169

[B30] Jeng YJ Kochukov M Watson CS Combinations of physiologic estrogens with xenoestrogens alter calcium and kinase responses, prolactin release, and membrane estrogen receptor trafficking in rat pituitary cells Environ Health 2010 9 61 10.1186/1476-069X-9-61 20950447 PMC2967504

[B31] Jeng YJ Watson CS Combinations of physiologic estrogens with xenoestrogens alter ERK phosphorylation profiles in rat pituitary cells Environ Health Perspect 2011 119 104 112 20870566 10.1289/ehp.1002512 PMC3018487

[B32] Wozniak AL Bulayeva NN Watson CS Xenoestrogens at picomolar to nanomolar concentrations trigger membrane estrogen receptor-alpha-mediated Ca2+ fluxes and prolactin release in GH3/B6 pituitary tumor cells Environ Health Perspect 2005 113 431 439 10.1289/ehp.7505 15811834 PMC1278483

[B33] Pappas TC Gametchu B Yannariello-Brown J Collins TJ Watson CS Membrane estrogen receptors in GH3/B6 cells are associated with rapid estrogen-induced release of prolactin Endocrine 1994 2 813 822

[B34] Pappas TC Gametchu B Watson CS Membrane estrogen receptor-enriched GH_3_/B6 cells have an enhanced non-genomic response to estrogen Endocrine 1995 3 743 749 10.1007/BF03000207 21153164

[B35] Bulayeva NN Gametchu B Watson CS Quantitative measurement of estrogen-induced ERK 1 and 2 activation via multiple membrane-initiated signaling pathways Steroids 2004 69 181 192 10.1016/j.steroids.2003.12.003 15072920 PMC1201430

[B36] Bulayeva NN Watson CS Xenoestrogen-induced ERK-1 and ERK-2 activation via multiple membrane-initiated signaling pathways Environ Health Perspect 2004 112 1481 1487 10.1289/ehp.7175 15531431 PMC1325963

[B37] Jeng YJ Kochukov MY Watson CS Membrane estrogen receptor-alpha-mediated nongenomic actions of phytoestrogens in GH3/B6/F10 pituitary tumor cells J Mol Signal 2009 4 2 10.1186/1750-2187-4-2 19400946 PMC2679742

[B38] Kochukov MY Jeng Y-J Watson CS Alkylphenol xenoestrogens with varying carbon chain lengths differentially and potently activate signaling and functional responses in GH_3_/B_6_/F10 somatomammotropes Env Health Perspect 2009 117 723 730 19479013 10.1289/ehp.0800182 PMC2685833

[B39] Campbell CH Bulayeva N Brown DB Gametchu B Watson CS Regulation of the membrane estrogen receptor-alpha: role of cell density, serum, cell passage number, and estradiol FASEB J 2002 16 1917 1927 10.1096/fj.02-0182com 12468456 PMC1266276

[B40] Jeng YJ Watson CS Proliferative and anti-proliferative effects of dietary levels of phytoestrogens in rat pituitary GH3/B6/F10 cells - the involvement of rapidly activated kinases and caspases BMC Cancer 2009 9 334 10.1186/1471-2407-9-334 19765307 PMC2755011

[B41] Vandenberg LN Colborn T Hayes TB Heindel JJ Jacobs DR Jr Lee DH Shioda T Soto AM Vom Saal FS Welshons WV Zoeller RT Myers JP Hormones and endocrine-disrupting chemicals: low-dose effects and nonmonotonic dose responses Endocr Rev 2012 33 378 455 10.1210/er.2011-1050 22419778 PMC3365860

[B42] Dufy B Vincent J-D Fleury H Pasquier PD Gourdji D Vidal AT Membrane effects of thyrotropin-releasing hormone and estrogen shown by intracellular recording from pituitary cells Science 1979 204 509 511 10.1126/science.107590 107590

[B43] Pappas TC Gametchu B Watson CS Membrane estrogen receptors identified by multiple antibody labeling and impeded-ligand binding FASEB J 1995 9 404 410 7896011 10.1096/fasebj.9.5.7896011

[B44] Schug TT Abagyan R Blumberg B Collins T Crews D DeFur P Dickerson S Edwards T Gore A Guillette LJ Hayes T Heindel J Moores A O’ Brien KP Patisaul HB Al TT Thayer K Vandenberg L Warner J Watson C Vom Saal FS Zoeller RT Myers JP Designing endocrine disruption out of the next generation of chemicals Green Chemistry 2013 15 181 198 http://pubs.rsc.org/en/journals/journal/gc 10.1039/C2GC35055F 25110461 PMC4125359

[B45] Jeng YJ Kochukov M Nauduri D Kaphalia BS Watson CS Subchronic exposure to phytoestrogens alone and in combination with diethylstilbestrol - pituitary tumor induction in Fischer 344 rats Nutr Metab (Lond) 2010 7 40 10.1186/1743-7075-7-40 20459739 PMC2881934

[B46] Conolly RB Lutz WK Nonmonotonic dose–response relationships: mechanistic basis, kinetic modeling, and implications for risk assessment Toxicol Sci 2004 77 151 157 14600281 10.1093/toxsci/kfh007

[B47] Watson CS Jeng YJ Kochukov MY Nongenomic signaling pathways of estrogen toxicity Toxicol Sci 2010 115 1 11 10.1093/toxsci/kfp288 19955490 PMC2902922

[B48] Weltje L vom Saal FS Oehlmann J Reproductive stimulation by low doses of xenoestrogens contrasts with the view of hormesis as an adaptive response Hum Exp Toxicol 2005 24 431 437 10.1191/0960327105ht551oa 16235731

[B49] Hunter T Protein kinases and phosphatases: the yin and yang of protein phosphorylation and signaling Cell 1995 80 225 236 10.1016/0092-8674(95)90405-0 7834742

[B50] McCubrey JA Steelman LS Chappell WH Abrams SL Wong EW Chang F Lehmann B Terrian DM Milella M Tafuri A Stivala F Libra M Basecke J Evangelisti C Martelli AM Franklin RA Roles of the Raf/MEK/ERK pathway in cell growth, malignant transformation and drug resistance Biochim Biophys Acta 2007 1773 1263 1284 10.1016/j.bbamcr.2006.10.001 17126425 PMC2696318

[B51] Allan LA Morrice N Brady S Magee G Pathak S Clarke PR Inhibition of caspase-9 through phosphorylation at Thr 125 by ERK MAPK Nat Cell Biol 2003 5 647 654 10.1038/ncb1005 12792650

[B52] Allan LA Clarke PR Phosphorylation of caspase-9 by CDK1/cyclin B1 protects mitotic cells against apoptosis Mol Cell 2007 26 301 310 10.1016/j.molcel.2007.03.019 17466630

[B53] Allan LA Clarke PR Apoptosis and autophagy: regulation of caspase-9 by phosphorylation FEBS J 2009 276 6063 6073 10.1111/j.1742-4658.2009.07330.x 19788417

[B54] Junttila MR Li SP Westermarck J Phosphatase-mediated crosstalk between MAPK signaling pathways in the regulation of cell survival FASEB J 2008 22 954 965 18039929 10.1096/fj.06-7859rev

[B55] Nordstrom E Fisone G Kristensson K Opposing effects of ERK and p38-JNK MAP kinase pathways on formation of prions in GT1-1 cells FASEB J 2009 23 613 622 18824519 10.1096/fj.08-115360

[B56] Xia Z Dickens M Raingeaud J Davis RJ Greenberg ME Opposing effects of ERK and JNK-p38 MAP kinases on apoptosis Science 1995 270 1326 1331 10.1126/science.270.5240.1326 7481820

[B57] Meloche S Pouyssegur J The ERK1/2 mitogen-activated protein kinase pathway as a master regulator of the G1- to S-phase transition Oncogene 2007 26 3227 3239 10.1038/sj.onc.1210414 17496918

[B58] Ip YT Davis RJ Signal transduction by the c-Jun N-terminal kinase (JNK)–from inflammation to development Curr Opin Cell Biol 1998 10 205 219 10.1016/S0955-0674(98)80143-9 9561845

[B59] Dhanasekaran DN Reddy EP JNK signaling in apoptosis Oncogene 2008 27 6245 6251 10.1038/onc.2008.301 18931691 PMC3063296

[B60] Sanchez-Perez I Murguia JR Perona R Cisplatin induces a persistent activation of JNK that is related to cell death Oncogene 1998 16 533 540 10.1038/sj.onc.1201578 9484843

[B61] Rhode PR Gorski J Growth and cell cycle regulation of mRNA levels in GH3 cells Mol Cell Endocrinol 1991 82 11 22 10.1016/0303-7207(91)90004-C 1761163

[B62] Bole-Feysot C Goffin V Edery M Binart N Kelly PA Prolactin (PRL) and its receptor: actions, signal transduction pathways and phenotypes observed in PRL receptor knockout mice Endocr Rev 1998 19 225 268 10.1210/er.19.3.225 9626554

[B63] Food and Drug Administration Drug Interaction Studies - Study Design, Data Analysis, Implications for Dosing, and Labeling Recommendations http://www.fda.gov/downloads/Drugs/GuidanceComplianceRegulatoryInformation/Guidances/UCM292362.pdf

[B64] Bulayeva NN Wozniak A Lash LL Watson CS Mechanisms of membrane estrogen receptor-{alpha}-mediated rapid stimulation of Ca2+ levels and prolactin release in a pituitary cell line Am J Physiol Endocrinol Metab 2005 288 E388 E397 10.1152/ajpendo.00349.2004 15494610

